# Semantic-Pragmatic Impairment in the Narratives of Children With Autism Spectrum Disorders

**DOI:** 10.3389/fpsyg.2019.02756

**Published:** 2019-12-12

**Authors:** Naama Kenan, Ditza A. Zachor, Linda R. Watson, Esther Ben-Itzchak

**Affiliations:** ^1^Department of Communication Disorders, Ariel University, Ariel, Israel; ^2^The Autism Center, Department of Pediatrics, Shamir Medical Center (Assaf Harofeh), Zerifin, Israel; ^3^The Sackler School of Medicine, Tel Aviv University, Tel Aviv, Israel; ^4^Department of Allied Health Sciences, School of Medicine, The University of North Carolina at Chapel Hill, Chapel Hill, NC, United States

**Keywords:** autism spectrum disorder, narrative performance, central ideas, *Tuesday* story, Autism Diagnostic Observation Schedule

## Abstract

Narrative impairments are common in autism spectrum disorders (ASDs). The Autism Diagnostic Observation Schedule (ADOS) battery includes a story-telling activity using a picture book called *Tuesday*. The current study aimed to identify differences between children with ASD and children with typical development (TD) on the production of *Tuesday* narratives, with a special focus on semantic-pragmatic aspects. Participants were 48 cognitively-able boys, in the age range of 4;10–7;0 years. Twenty-four participants were boys with ASD and 24 participants were TD boys. The semantic-pragmatic analysis included measures of: story details (characters setting, objects, and actions), central ideas, evaluative comments, and unrelated text. Results showed that the narratives produced by children with ASD included fewer central ideas, and fewer settings, characters, and actions, but not objects, as compared with the narratives produced by their TD peers. The number of evaluative comments and utterances that were unrelated to the story did not differ between the groups. A negative correlation was found between the autism severity level and the number of central ideas and number of characters mentioned in the narratives of the ASD participants. Taken together, as a group, these findings point to a semantic-pragmatic impairment in ASD. However, individual analysis revealed heterogeneity within the ASD group in this area. Some of the results may be explained by cognitive deficits in maintaining central coherence (the Weak Central Coherence account). This study has important clinical implications. Defining the specific differentiating measures can maximize the use of the ADOS story-telling activity by clinicians. The association found between the autism severity level and some of the semantic measures can be used in evaluating the severity of the ASD symptoms.

## Introduction

Autism spectrum disorder (ASD) describes a wide range of symptoms. The core symptoms in ASD are defined as a difficulty with social-communication skills and restricted interests and repetitive behaviors (RRBs) (American Psychiatric Association [APA], 2013). In addition to the core symptoms, some individuals with ASD show specific deficits in cognition (Grzadzinski et al., 2013) and language (Harper-Hill et al., 2013). In both domains, children with ASD comprise a highly heterogeneous group with great variability. Recent research reported that 46% of children with ASD were in the average or above average range of intellectual ability (IQ > 85) (Centers for Disease Control and Prevention [CDC], 2015). With respect to verbal ability in ASD, a wide range of linguistic competence is described in the literature, from a total lack of verbal language to fluent and even advanced language skills (Harper-Hill et al., 2013). However, even among individuals with ASD whose cognitive and language achievements in standardized assessments are within normal limits, pragmatic and semantic impairments are still commonly reported (Tager-Flusberg et al., 2005).

### The Bases of Narrative Skills

Narrative discourse is an important skill, through which children organize their experiences and communicate them to others (Bruner, 1991). It plays a significant role in social interactions and in academic performance (Berman and Slobin, 1994). Narrative competence relies on a diverse range of linguistic and cognitive skills. In terms of language, it is considered a “high-order” task, in which all aspects of language play a significant role. In addition, coherent narratives require the ability to link a series of events and actions according to temporal and causal principles. In terms of cognition, the comprehension and production of narratives requires a variety of capabilities, such as perceiving the gestalt of a complex stimulus, inferring abstract ideas from concrete details, and taking into account the perspective of the listener, known as theory of mind (ToM). Because of the numerous capacities inherent in narrative production, the analysis of narratives produced by children with TD and children with developmental disorders has become a widely used measure of communicative and cognitive abilities. In ASD, the analysis of narratives is especially relevant, since many of the prerequisites for narrative production are affected. Narrative analysis provides an excellent means of looking into the core cognitive and linguistic bases of this disorder. Consequently, narrative production has been studied extensively in individuals with ASD (for review Stirling et al., 2014; Baixauli et al., 2016).

Three of the leading theoretical accounts that attempt to explain the core deficits in ASD are: the Weak Central Coherence (WCC) account (Happé, 1999; Frith, 2003; Happé and Frith, 2006), ToM deficit (Baron-Cohen et al., 1985; Tager-Flusberg and Sullivan, 1995) and executive function (EF) impairment (Hill, 2004; Kenworthy et al., 2008). These theoretical accounts can be linked directly to the narrative performance of children with ASD. According to the WCC account, the cognitive style of individuals with ASD reflects a tendency to favor processing of local stimulus properties at the expense of integrating details into meaningful wholes. At the “low” level of information processing, they tend to neglect context in the sensory (e.g., visual, acoustic) domain, and at the “high” level of information processing, they demonstrate difficulties with more abstract, conceptual processes (Frith and Happé, 1994). The WCC cognitive style exhibits difficulties in using context to infer meaning (Vermeulen, 2015). When pictures are used to elicit narrative production, it may affect the comprehension of concepts that need to be inferred from the integration of several visual details. According to the ToM account, people with ASD show a deficit in their ability to infer other peoples’ mental states. In regards to narrative capacity, ToM impairment may challenge their ability to take into account the listener’s previous knowledge. They may also fail to identify and relate to the psychological states of the story’s characters, including their perceptions, emotions, and motivations (Tager-Flusberg and Sullivan, 1995; Capps et al., 2000). The EF deficiency account focuses on the difficulties that individuals with ASD often demonstrate in performing a variety of complex tasks, such as planning, self-monitoring, inhibition, and mental flexibility (Ozonoff et al., 1991). There is evidence that the development of EF skills and the development of narrative skills support each other (e.g., Friend and Bates, 2014). EF weakness may impact narrative competence in several different ways, such as in difficulty organizing information regarding the content of the story, and securing a connection between sentences. It may also reduce the ability to self-monitor performance throughout narration and to focus on the significant information while omitting irrelevant details (Joseph and Tager-Flusberg, 2004; Ketelaars et al., 2012).

### Narrative Skills in ASD

Previous studies on the narrative skills of children and adults with ASD have yielded inconsistent results (Baixauli et al., 2016). This inconsistency may be due to differences in the definition of the research groups (high vs. low functioning individuals), general language function (at or below age norms), ranges of chronological age, matching criteria (chronological age, mental age, or language function), and story elicitation procedures (story retell, telling a personal experience, or picture book narration). Even when cognitively-able children with ASD were matched to controls on their overall language level, some studies found group differences on some of the measures, but others could not differentiate between the groups [see Supplementary Material (Table A1), for summary of selected studies]. There are findings that point to difficulty in maintaining coherence (Norbury and Bishop, 2003; Diehl et al., 2006) including incorrect use of pronouns (Norbury and Bishop, 2002; Novogrodsky, 2013), and addition of irrelevant information (Losh and Capps, 2003; Makinen et al., 2014). Other findings point to a difficulty in accurately conveying the semantics of the story in a pragmatic way. For the purposes of this essay, the semantics of the story and the ways by which it is conveyed in the narratives will be referred to as “semantic-pragmatic” aspects of the narrative. For example, children with ASD were found to relate to causal aspects of the story less often than their TD peers (Losh and Capps, 2003; Sah and Torng, 2015). Children with ASD were also found to include fewer main story components and to make fewer references to internal states and their causes as compared with their TD controls (Makinen et al., 2014). In a meta-analysis conducted by Baixauli et al. (2016), it was found that children with ASD, without intellectual disability, performed significantly worse than their peers on different measures of narrative length, syntactic complexity, coherence, cohesiveness, and Internal State Language (ISL).

### Research on the *Tuesday* Narratives (ADOS) in ASD

Evaluating narrative ability is part of the observational assessment for diagnosing ASD, the Autism Diagnostic Observation Schedule (ADOS) (Lord et al., 1999). Module 3 of the ADOS is designed for use with children and adolescents who have fluent language skills. It includes an activity of telling a story from a wordless picture book called *Tuesday* (Wiesner, 1991). The story is about the adventures of a colony of frogs, which magically start flying over water lily pads, and take a tour of a nearby town. The purpose of this activity is to assess the participant’s ability to understand and tell a sequential story from a book of pictures. This task provides a context for the child to comment on social relationships and affect. The focus of this section is on obtaining a sample of the participant’s spontaneous language and communication, gaining an understanding of what captures his/her interest, and evaluating the child’s interpretation of visual indications of social contexts (Lord et al., 2000). In order to produce an adequate narrative, one has to refer to a variety of concrete concepts as well as to a series of abstract ideas.

Several studies have compared the narrative ability of children with ASD and their typically developing (TD) peers using the *Tuesday* picture book from Module 3 of the ADOS (Rumpf et al., 2012; Banney et al., 2014; Suh et al., 2014; Kauschke et al., 2016; Kuijper et al., 2016) [see Supplementary Material (Table A2)]. The participants in these studies varied in their age ranges (from 6;0 to 19;0), but all were high functioning (HF) children with ASD (IQ scores above 70), and their TD peers, who were matched to the research group on chronological age, gender, non-verbal cognition, and overall language proficiency. A variety of measures have been used in these studies. As can be seen in Supplementary Material (Table A2), once again, results are inconsistent regarding all types of measures: narrative productivity (i.e., story length or sentence length), fluency measures (repetitions, self-corrections, and fillers), morphosyntactic skills, pronoun use, idiosyncratic language, cohesive devices (e.g., causal connectors), evaluation comments, and personal style. One variable that was consistently found to be comparable between the groups was lexical diversity, suggesting that HF children with ASD have lexicons that are just as diverse as their age-matched TD peers.

The semantic-pragmatic analysis conducted in previous *Tuesday* studies focused on the use of ISL, and on the story’s main details and events. The analysis of ISL yielded group differences in Rumpf et al. (2012) and Kauschke et al. (2016) who compared female individuals with ASD to TD females and found group differences for terms of emotion. The ASD participants in the three other studies evaluating ISL (Banney et al., 2014; Suh et al., 2014; Kuijper et al., 2016) produced a similar number of these terms as the TD participants. When the story’s basic components were analyzed, Rumpf et al. (2012) and Kauschke et al. (2016) found no group difference in the overall reference to the story’s characters, time, and space. In addition, Kauschke et al. (2016) did not find a group difference in relation to the two examined “core events” (“frogs are not able to fly in reality” and “the frogs lose their capability to fly on water lily leaves; the policemen wonder where the leaves on the street come from”). However, Suh et al. (2014) found that their ASD group included significantly fewer story elements (a pre-identified list of the main events of the story) than the TD group. Likewise, Rumpf et al. (2012) found that their ASD participants verbalized the two examined “core events” of the story (i.e., “frogs lose their ability to fly,” and “the police are wondering where the leaves came from”) significantly less than their TD peers. Finally, Banney et al. (2014) who also examined only two core events, found that ASD participants included them less in their narratives: “the emotional response of the frogs” and “the frogs lose their ability to fly.” Taken together, these results point to a possible semantic-pragmatic difficulty in the narrative production of children with ASD. The term “semantic-pragmatic,” in this study, is used to refer to the process of generating and conveying meaning within social and interpersonal context. However, the analysis of the story’s events and ideas conducted in previous studies was limited in its scope. Suh et al. (2014) did analyze a list of the story’s events; however, the majority of the events were concrete descriptions of the pictures and there was no differentiation between those events and the more globalized and abstract meanings.

### Rationale and Hypotheses

The *Tuesday* story gives an excellent conceptual framework for semantic-pragmatic analysis. In addition to many basic, concrete details, such as characters, settings, objects, and events, it contains several unique ideas. These ideas will be referred to, from now on, as *central ideas*. They require the integration of details into global meanings, and the ability to infer implicit and abstract concepts from apparent information. Some ideas require reference to the characters’ emotional and cognitive states, and some require the interpretation of social contexts. In contrast to *events*, as analyzed in previous studies, and *actions*, as analyzed in the current study, *central ideas* require interpretation beyond the concrete visual stimuli and the ability to relate to information conveyed in previous pictures. Thus, the main goal of the current study was to identify the specific semantic-pragmatic measures that differentiate the *Tuesday* narratives of children with ASD from the narratives of their TD age-matched controls, at both the concrete and the abstract, global levels. Since semantics-pragmatics is a core area of impairment in the language of children with ASD, we were interested in determining whether there is a correlation between the semantic-pragmatic skills of children with ASD, as expressed by their ability to relate to the story’s central ideas, and their autism severity level. The participants in this study were comprised of children in the youngest age range specified for using the *Tuesday* story, 4;10–7;00 (Module 3 is designed for children and adolescents who have fluent language skills, estimated at a 4-year-old level in functional expressive language or higher). This is the first study in which children under 6 years of age have been examined.

Based on the cognitive theories of ASD and previous studies, we hypothesize that children with ASD will show a semantic-pragmatic deficit, as expressed in fewer references to the story’s basic details and central ideas. The deficit will arise specifically in relation to concepts that require the integration of several pieces of information and/or consideration of the story’s context. These concepts include the story’s central ideas and the story’s basic details, except for the category of objects, which is more concrete in nature.

The study has both theoretical and clinical significance. Theoretically, examining the differences in narrative ability between children with ASD and children with TD may highlight the neurocognitive atypicalities that underlie the core deficits of ASD. In relation to the three cognitive accounts of ASD, a finding that children with ASD refer less to the story’s main ideas, as compared with their TD controls, may support the WCC theory. A finding that children with ASD refer less often to the characters’ mental states as compared with their TD controls may support the ToM account of ASD. A finding of more associative, unrelated utterances in the narratives of children with ASD, as compared with their TD controls, may support an EF deficiency in ASD.

Clinically, defining the specific differentiating measures can maximize the use of the ADOS story-telling activity, and help clinicians in the ASD diagnosis procedure. In addition, the study aimed to characterize the normative performance of children with TD on the *Tuesday* story, in the examined age range, in order to give clinicians clear and structured guidelines for comparison with children who have ASD.

## Materials and Methods

### Participants

The participants in the study were 48 males, in the age range of 4;10–7;0 (*M* = 6;3 years, *SD* = 5 months). Of the study population, 24 boys were diagnosed with ASD (the research group) and 24 boys were TD participants (the control group). The reason for using only male participants was the small number of female subjects who were available within the chosen age range, in accordance with the reported male to female ratio in ASD (Loomes et al., 2017). By using only male subjects we ensured a highly homogenous group. The research group was selected from a population that underwent a comprehensive evaluation for ASD at a tertiary autism center. The evaluation included neurological, behavioral, cognitive, and functional assessments, which were conducted by a skilled interdisciplinary team. The diagnosis of ASD was confirmed using two standardized tests, the Autism Diagnostic Interview-Revised ADI-R (Le Couteur et al., 2003) and the ADOS (Lord et al., 1999). All the professionals involved in the diagnostic process established reliability in the ASD diagnostic tools as required. Cognitive abilities (IQ) were assessed using the Wechsler Preschool and Primary Scale of Intelligence- Third Edition (WPPSI-III) (Wechsler, 2002). The criteria for selection of the participants included: being a male in the defined age range, having verbal and non-verbal scores above 80 on the WPPSI-III (Wechsler, 2002), and meeting cutoff criteria for ASD on at least one of the standardized ASD evaluations: the ADI-R (Le Couteur et al., 2003) or the ADOS Module 3 (Lord et al., 2000). All the participants in the research group received a final clinical diagnosis of ASD based on the Diagnostic and Statistical Manual of Mental Disorders (DSM)-IV or V (American Psychological Association (American Psychiatric Association [APA], 2000, 2013) criteria, depending on the time of diagnosis. All ASD participants attended regular kindergarten or schools in accordance with their age.

The TD male participants were recruited from local, regular education kindergartens and schools and were pair-matched to the ASD group according to age (up to ± 4 months age difference). A criterion for inclusion in the control group was a standard score of > 7 on the Vocabulary (verbal) and Blocks Design (non-verbal) WPPSI-III subtests. As shown in Table 1, the two groups did not differ in their mean chronological age, or in their scores on the Block Design and Vocabulary cognitive subtests, using analysis of variance (ANOVA) and Multivariate Analysis of Covariance (MANCOVA) respectively. The groups differed in their average parental education. Parents of the TD group were found to have a higher overall educational attainment. According to the ASD participants’ medical files, three participants had one parent born in Israel and the other in a different country (in France, Germany, and the United States), and all other participants had two parents born in Israel. Although direct information on the language status of the ASD participants was not available, it appears that 88% of them were monolingual, Hebrew native speakers. All TD participants were monolingual, native Hebrew speakers, based on parental reports. However, data regarding their exposure to a second language in their home environments were not available. All procedures performed in this study were in accordance with the ethical standards of the institutional and/or national research committee and with the 1964 Helsinki Declaration and its later amendments or comparable ethical standards. This research was approved by the Helsinki Committee at the Shamir Medical Center (Assaf Harofeh) and by the Institutional Review Board (IRB) of the University as required. Since data for the ASD participants was based on their medical charts, the IRB committee released the researchers from having a parental consent for this group. Informed consent was obtained from all parents of TD participants included in the study.

**TABLE 1:** Characteristics of participants.

	**ASD group Range; M (SD)**	**TD group Range; M (SD)**	***F***	***p***	**ω^2^**
Age (years; months)	4;10–7;2 6;4 (0;7)	4;11–7;2; 7;7 (0;7)	0.1	0.79	0.00
Block design subtest	7–18; 11.7 (2.9)	8–15 11.4 (2.4)	0.1	0.71	0.00
Vocabulary subtest	6–17; 10.7 (2.6)	7–16 11.7 (2.5)	1.6	0.21	0.01
IQ scores	83–135; 100.7 (11.3)	NA	–		–
Verbal IQ scores	81–135; 102.7 (13.2)	NA	–		–
Non-verbal IQ scores	84–137; 104.2 (11.8)	NA	–		–
ADOS-CSS	4–10; 7.7 (1.8)	NA	–		–
ADOS-SA-CSS	3–10; 7.0 (2.2)	NA	–		–
ADOS-RRB-CSS	1–10; 7.8 (2.6)	NA	–		–
Maternal education	12–18; 14.7 (2.2)	12–21; 16.1 (1.9)	**5.5**	0.02	0.09
Paternal education	11–18; 14.3 (2.5)	12–19; 15.9 (1.8)	**6.6**	0.01	0.11

*All measures of IQ are standard scores. Bold values are statistically significant.*

### Procedure

Data for the ASD group, including age, IQ scores, parental education, ADOS calibrated severity scores (CSSs) and final diagnosis, were obtained from medical charts. The children underwent a comprehensive assessment that included behavioral, cognitive, and adaptive skill assessment. The assessment was conducted by a skilled interdisciplinary team. The professionals who performed the ADOS established reliability in this standardized test as required. The parents of TD participants filled out a questionnaire relating to early development and biographical details, which was used to verify typical development (TD). The TD participants underwent evaluation of two subtests of the WPPSI-III, Vocabulary (verbal) and Blocks Design (non-verbal), as a screening procedure of cognitive function. These two subtests are considered to be representative of verbal and non-verbal abilities (Hrabok et al., 2014).

All participants were given the *Tuesday* picture book of the ADOS Module 3, and were asked to tell a story accordingly. The participants with ASD completed this task as part of their diagnostic procedure at the autism center. The TD participants were administered the task in a quiet room either at their kindergartens/schools or in their homes by two SLP students who were in the final stage of their studies in the Communication Disorders Department. For the purpose of the current study, the narration of the *Tuesday* story was transcribed from videotapes of the ADOS procedure by a certified speech and language pathologist (SLP). The narratives of the TD participants were audio recorded and transcribed by the SLP students who administered the task. An identical set of instructions was given to each participant, with the examiner describing the first picture in the book, and then asking the child to continue telling the story with the subsequent pictures. It is worth noting that children in both groups did not see the pictures before telling the story, in accordance with the ADOS manual. In both participant groups, examiners attempted to intervene minimally in the child’s story, and tried to limit their comments to general prompting such as: “Tell me more” or “What happened next?” However, some children needed more specific prompting in order to keep the story going and in order to clarify unclear text. These prompts were later counted and compared between the groups, as will be described in Section “Measures.”

The high-quality recordings of both groups were transcribed according to the accepted clinical guidelines. Narration of each picture in the book was transcribed separately. Utterances ranged from one word expressions to complete sentences (simple, coordinated, or conjoined). All transcripts were coded according to a set of pre-determined measures, and included a semantic-pragmatic coding system that was developed especially for the *Tuesday* story, as described in detail in the next section.

### Measures

#### Autism Diagnostic Observation Scales (ADOS)

It is a semi-structured, interactive schedule designed to assess social and communicative functioning in individuals who may have ASD. Only one of the modules is administered, depending on the examinee’s age and/or expressive language (Lord et al., 1999, 2000). ASD participants for the current study were all administered module 3, which is designed for children who use fluent expressive language. The scores of each of the ADOS sub-domains, social affect (SA) and RRBs were used for the calculation of each sub-domain severity score using the SA and RRB-CSS. Scores from the original ADOS protocol were converted to compute the cut-offs (SA, RRB, and subsequent CSS scores) from the ADOS-2 algorithms. The range for ADOS-CSS and ADOS-SA-CSS is 0–10. The range for ADOS-RRB-CSS is 5–10 (this measure does not include scores between 1 and 4). Higher scores reflect more severe autism symptoms for each domain (Hus et al., 2014).

#### Wechsler Preschool and Primary Scale of Intelligence- Third Edition

An intelligence test designed for children ages 2 years and 6 months to 7 years and 7 months (Wechsler, 2002). It provides subtest and composite scores that represent intellectual functioning in verbal and performance cognitive domains, as well as providing a composite score that represents a child’s general intellectual ability (i.e., Full Scale IQ).

#### Narrative Analysis

The 15-page picture book *Tuesday* begins with frogs sitting on lily pads in a pond during sunset. Magically, they rise into the air and start flying. When they enter a nearby town they experience an array of events and adventures. As day breaks, the frogs lose their ability to fly, fall down to the earth, and hop back to their pond. The next morning, police officers try to figure out why lily pads are found all over town. The following *Tuesday*, the magic repeats itself, and pigs start flying. The current analysis included three types of measures: (1) administration conditions: prompting; (2) linguistic measures which were used to confirm identical structural language abilities among the groups: overall story length and syntactic complexity; and (3) the focus of the current study: semantic-pragmatic measures: story details, central ideas, evaluative comments, and unrelated text.

##### Prompting

Since the task was administered to participants in two different settings and by different examiners, we measured the number of comments made by the examiners during the storytelling, which may have affected the inclusion of specific story details by participants in their narratives. All cases in which the examiner intervened during the task with specific remarks or questions that directed the child’s attention to a specific detail in the story were counted.

##### Overall story length

The quantitative measure chosen to reflect the overall length of the produced narrative was the number of clauses, in simple sentences (one independent clause), in compound sentences (two independent clauses or more), and in complex sentences (at least one independent clause and at least one dependent clause). Utterances that were “yes/no” responses, reflections on the story (e.g., “this is a long story”), and unintelligible sentences/clauses, were excluded from this count.

##### Syntactic complexity

In order to measure the participants’ linguistic performance, the percentage of subordinate clauses out of the total number of clauses, either within a complete, complex sentence or as separate clauses that were produced pragmatically (e.g., in response to a question), was calculated. In addition, percentages of morphological and syntactic errors were calculated.

##### Story details

This part of the semantic-pragmatic analysis related to the basic and mostly concrete story components. Most of these concepts required the direct interpretation of visual stimuli (the pictures). They included settings, objects, characters, and actions. A list of these essential items was prepared in advance for each category. Only details that were included spontaneously and accurately in the narrative by each participant were counted. The list included:

*Settings* (total = 6): swamp/pond/pool, city/houses, house/kitchen, garden/yard, house/living room of the old lady, street/road.

*Objects* (total = 8): leaves/water lilies, electric pole, sandwich/supper, laundry/sheets/blankets, window/chimney, fireplace, television/remote control, cars/trucks.

*Characters* (total = 7): frogs, birds, man/father/husband, grandmother/lady/old lady, dog, policemen/officers/investigators, and pigs.

*Actions* (total = 11): this measure focused on the use of specific verbs which describe the visible actions of the characters in the story: frogs flying, man eating, frogs encountering the laundry, frogs entering the house, grandma sleeping, frogs watching television, frogs running away from dog, frogs chasing dog, frogs falling down, policemen checking/viewing the scene.

*Central ideas***:** This part of the semantic-pragmatic analysis attempted to capture the ability of children to comprehend and expressively relate to the unique set of ideas that comprise the *Tuesday* story. The list of ideas was compiled by the researchers, by going through the pictures and defining the main central idea for each page. In a pilot study we analyzed the *Tuesday* narratives of 39 neurologically intact adults, and we selected the ideas that were referred to by more than 40% of these participants (Kenan et al., 2018). These ideas require abstraction, inferencing, integration of details, and ToM skills. For example, in order to formulate central idea no. 7 = “the changing role of the dog,” the child needs to integrate the details from two consecutive pictures in which the direction of movement is reversed. Central idea no. 8 = “frogs falling down as a result of the magic’s ending” requires the understanding that their flight was magical, that the lily pads served as their “magic carpets,” and that the magic’s ending led to their falling down. Four out of 12 central ideas were related directly to ToM, and were defined as such for later analysis (no. 2, 3, 5, and 10). Only ideas that were included in the narratives spontaneously and accurately were counted for each participant. The ideas were:

(1) Frogs are flying over lily pads;
(2) Thoughts of the turtle (or birds) (ToM);
(3) Thoughts of the man eating supper (ToM);
(4) Frog is making a gown/apron/parachute out of sheets/blankets;
(5) The old lady is sleeping and not aware of the frogs visiting her home (ToM);
(6) Frog is switching channels on the T.V. with its tongue;
(7) The changing role of the dog – first chasing the frogs, then being chased by them;
(8) Frogs falling down as a result of the magic’s ending;
(9) Policemen are investigating the events;
(10) Police officer is puzzled by the leaves (ToM);
(11) The changing time – evening-night-morning;
(12) Pigs flying like the frogs.

The categorization of central ideas into ToM and non-ToM-related was based on the clinical and research experience of the first and last authors. However, in order to verify this categorization, an inter-rater reliability procedure was used. Twenty-one raters (students who were in the last stage of their studies in the Communication Disorders Department) were asked to categorize central ideas that require ToM skills. Judgments reached over 85% agreement with the original categorization of the authors.

##### Evaluation

This variable captured the personal perspective of the narrator, and included: emotions/opinions of the narrator toward the story, adjectives, adverbs, and discourse markers (e.g., “finally”), and modal (e.g., “should”), aspectual (e.g., “began”), emotional (e.g., “was scared”), cognitive (e.g., “knew”), and perceptual (e.g., “heard”) verbs. All of these “evaluative” elements were counted for each participant.

##### Unrelated text

This variable attempted to quantify the portion of the narrative that was unrelated to the story’s semantic content. We counted all utterances which were associative, such as: “The man was afraid of the frogs so he went to his bedroom and found more animals in there too…,” which was the description given by one child with ASD about the picture of the man who was eating supper while watching the frogs flying outside his window. This measure also included incorrect interpretations of pictures, such as: “The frog is licking the book…” which was the description given by another child with ASD for the picture in which the frog was using its tongue to switch channels on the old lady’s TV set.

Sample transcripts of one participant with ASD and one participant with TD are provided in Supplementary Material (Appendix B), including highlighting of the central ideas mentioned in the text.

### Data Analysis

Group means of participants’ characteristics (e.g., age, cognitive subtest scores) and all continuous variables (e.g., number of *settings*, *characters*, central ideas) were compared between the groups using one-way ANCOVAs or MANCOVAs. Since parental education was found to be higher for the control group, it was entered as a covariate to all comparisons. For effect sizes, omega squared values were used (0.01 = small effect; 0.06 = medium effect; >0.14 = large effect). All continuous dependent variables in the study were examined for normality of distribution in each of the examined groups. The number of prompts (for the ASD group), the percentage of morphological errors and subordinate clauses (for both groups), and the number of evaluative comments (for both groups) did not show normal distribution as Skewness and Kurtosis values were not within the range of −1.96 to +1.96 (Kim, 2013). All other variables showed normal distributions in both groups according to this criterion. To avoid possible multicollinearity between continuous dependent variables, Pearson correlation analyses were performed between these measures. All correlations ranged from 0.01 to 0.61 (<0.7), therefore multicollinearity was ruled out for the examined variables. For the variables with non-normal distribution, non-parametric Mann–Whitney tests were added to the analyses. For categorical variables (e.g., percentage of participants who included each special idea in their narratives) non-parametric tests were used. In addition, correlations were performed between the semantic-pragmatic measures and measures of autism severity (ADOS–CSS-SA, ADOS–CSS-RRB) using Pearson correlation analyses.

### Reliability

All narratives were analyzed and coded by two of the study’s authors. To test for inter-examiner reliability of the transcription procedure, five transcripts (10.4%) of the recordings were transcribed by two of the authors. Reliability rating was found to be 92%. In order to test for inter-examiner reliability for the analysis procedure, 10 narratives (20.8%) were analyzed separately by the first and last authors. The last author was blind to the children’s diagnoses. An agreement of over 85.0% on all measures was achieved (percentage of concordantly transcribed tokens).

## Results

### Administration Conditions

#### Prompting

In order to confirm identical task administration for the two groups, the mean number of prompt**s** was compared using a one-way ANCOVA (see Table 2). No significant difference was found between the ASD and the TD groups. In addition, a non-parametric Mann–Whitney test was performed and did not yield a significant group effect (*Z* = −1.5, *p* = 0.13). The result shows that children with ASD were as autonomous in the task as TD children. In addition, this finding confirmed identical task administration conditions for the two groups.

**TABLE 2:** Analysis of narrative measures.

	**Range**	**ASD M (SD)**	**TD M (SD)**	***F***	***p***	**ω^2^**
Number of prompts	0–29	10.1 (6.5)	7.2 (5.5)	3.1	0.08	0.04
**Linguistics measures- (MANCOVA I)**						
Number of clauses (length)	9–57	40.9 (12.4)	31.6 (8.8)	7.5	**0.01**	0.12
Grammatical errors (%)	0–25.7	6.0 (5.8)	2.5 (3.4)	4.7	**0.04**	0.07
Complex sentences (%)	3.2–32.0	17.3 (8.3)	10.4 (6.2)	6.6	**0.01**	0.10
**Story details (MANCOVA II)**						
Settings	0–4	1.4 (1.1)	2.2 (1.0)	6.8	**0.01**	0.11
Objects	0–5	2.6 (1.2)	3.3 (1.4)	1.6	0.21	0.01
Characters	2–7	4.8 (1.2)	5.7 (1.3)	6.7	**0.01**	0.11
Actions	1–11	4.7 (1.8)	6.9 (2.2)	10.7	**0.00**	0.17
**Central ideas (MANCOVA III)**						
ToM ideas	0–4	1.2 (0.6)	1.9 (1.1)	5.4	**0.02**	0.09
Non-ToM ideas	0–4	0.9 (0.7)	1.6 (1.1)	4.2	**0.05**	0.06
Evaluative comments	0–25	17.7 (5.3)	16.9 (6.6)	0.00	0.98	0.00
Unrelated text (%)	0–22	11.7 (7.3)	7.8 (6.2)	2.7	0.10	0.03

*Bold values are statistically significant.*

#### Linguistic Measures

To assess linguistic abilities in overall story length, the percentage of errors and percentage of complex sentences were compared between the groups using a one-way MANCOVA. The analysis yielded a significant group effect [*F*(3,43) = 7.00, *p* = 0.001 ω^2^ = 0.28]. Separated one-way ANCOVAs for each dependent variable revealed significant group effect for the three measures. For the Overall story length, as presented in Table 2, the TD group had significantly more clauses than the ASD group. For the Syntactic Complexity measure, the ASD group had a significantly higher percentage of complex sentences than the TD group (Table 2). In addition, the ASD group had a significantly higher percentage of errors than the TD group, but both groups had a generally low percentage of errors overall. Finally, the non-parametric Mann–Whitney tests which were performed confirmed these results as they yielded significant group effects for the percentage of complex sentences (*Z* = −3.0, *p* = 0.00) and the percentage of grammatical errors (*Z* = −2.5, *p* = 0.01).

### Semantic-Pragmatic Measures

#### Story Details

First, the basic story details, including settings, objects, characters, and actions (Table 2) were analyzed. The total number of details mentioned in the narratives for each category was compared. The one-way MANCOVA yielded a significant group effect [*F*(4,43) = 4.2, *p* = 0.006, ω^2^ = 0.21]. Examining the specific variables revealed that for the number of settings, characters and actions, the TD group included significantly more details than the ASD group. No significant group difference was found for the number of objects (Table 2).

#### Central Ideas

The MANCOVA for ToM and non-ToM central ideas yielded a significant group effect [*F*(2,44) = 3.5, *p* = 0.04, ω^2^ = 0.10]. Examining each type of idea separately revealed that the TD group referred to more ToM and non-ToM ideas than the ASD group did (Table 2).

For each one of the central ideas, the percentage of participants who expressed them in their narratives was compared between the groups (Table 3). Two central ideas: “Frogs are flying over lily pads” (no. 1), and “The changing role of the dog” (no. 6) yielded significant group differences, in favor of the TD group. Only *special idea* no. 7 was included by more than 70% of the TD participants.

**TABLE 3:** Comparison of percentages of participants in the ASD and TD groups who included each of the central ideas in their narratives.

**Central ideas**	**% ASD**	**% TD**	**χ^2^**	***p***
(1) Frogs are flying over lily pads	8.3	50.0	**10.1**	0.00
(2) Thoughts of the turtle (or birds)	12.5	33.3	2.9	0.07
(3) Thoughts of the man eating supper	20.8	12.5	0.6	0.44
(4) Frog is making a gown/apron/parachute out of the sheets/blankets	33.3	58.3	3.0	0.08
(5) The old lady is unaware of the frogs visiting her home	25.0	33.3	0.4	0.52
(6) Frog is switching channels on the T.V. with her tongue	25.0	29.2	0.1	0.74
(7) The changing role of the dog = chaser & chasee	45.8	79.2	**5.7**	0.02
(8) Frogs falling down as a result of the magic’s ending	0.0	0.0	–	–
(9) Police are investigating the events	13.0	33.3	2.9	0.09
(10) Police officer is puzzled by the leaves	12.5	20.8	0.6	0.44
(11) New magic with pigs flying	8.3	0.0	2.1	0.15
(12) The changing time – evening-night-morning	0.0	0.0	–	–

*Bold values are statistically significant.*

We then organized the number of central ideas expressed in the narratives into four ranges (0, 1–2, 3–4, 5–8), and compared the percentage of participants in the two groups whose number of expressed central ideas was within each range. As can be seen in Table 4, a quarter of the TD group expressed 5–8 central ideas, while none of the ASD participants expressed more than four ideas. In contrast, more than half of the ASD group and only a quarter of the TD group expressed 1–2 ideas. These differences were statistically significant.

**TABLE 4:** Comparison of percentages of participants in the ASD and TD groups who included different ranges of central ideas in their narratives.

**Ranges of central ideas**	**ASD**	**TD**	**χ^2^**	***p***
0	8.3%	0%	2.1	0.15
1–2	54.2%	25.0%	**4.3**	0.04
3–4	37.5%	50%	0.762	0.38
5–8	0%	25%	**6.9**	0.01

*Bold values are statistically significant.*

#### Evaluative Comments

We compared the percentage of evaluation expressions (out of the total length) between the ASD and the TD groups using a one-way ANCOVA. No significant group effect was found (Table 2). In addition, a non-parametric Mann–Whitney test was performed and did not yield a significant group effect (*Z* = −1.1, *p* = 0.29).

#### Unrelated Text

The mean percentage of unrelated utterances (out of the total length) was compared between the groups using a one-way ANCOVA. No significant difference was found between the ASD and the TD groups (Table 2).

It should be noted that in all the described analyses, parental education did not yield a significant effect.

### Correlations of Semantic-Pragmatic Measures and ASD Severity

To examine the relationship between semantic-pragmatic measures and ASD severity, a correlation between these items and the ADOS autism severity levels was conducted, using Pearson correlation tests. As shown in Table 5, the ADOS-CSS-SA correlated negatively and significantly with the number of central ideas and showed a statistical trend toward characters. None of the semantic-pragmatic items correlated significantly with the ADOS-CSS-RRB.

**TABLE 5:** Pearson correlations of semantic-pragmatic items with autism severity measures (ADOS-CSS-SA, ADOS-CSS-RRB).

	**Settings**	**Characters**	**Actions**	**Central ideas**
ADOS-CSS-SA	−0.22	−0.31^	−0.20	−0.38^∗^
ADOS-CSS-RRB	−0.03	0.02	−0.09	0.12

*^∗^*p* < 0.05, ^*p* < 0.1.*

### ASD Sub-Groups According to Narrative Semantic-Pragmatic Ability

Since not all the children with ASD showed difficulty in relating to the central ideas of the *Tuesday* story relative to their TD controls, we decided to conduct an individual analysis. We looked at the number of central ideas for each participant with ASD in relation to the mean central ideas of the TD group. Accordingly, we divided the ASD group into two sub-groups with high and low semantic-pragmatic skills. All ASD participants whose mean number of central ideas fell one SD or more (0–2) below the mean of the TD group were considered to have “low narrative semantic-pragmatic skills.” Examining the ADOS-SA-CSS revealed higher scores for the “Low semantic-pragmatic skills group” (*n* = 15; *M* = 7.5, *SD* = 2.1) than the “High semantic-pragmatic skills group” (*n* = 9; *M* = 6.2, *SD* = 2.3). However, this difference did not reach statistical significance [*F*(1,22) = 2.1, *p* = 0.16, ω^2^ = 0.04). For the ADOS-RRB-CSS, no significant group effect was noted [*F*(1,22) = 0.31, *p* = 0.58, ω^2^ = 0.00) (“Low semantic-pragmatic group” : *M* = 7.6, *SD* = 2.5) (“High semantic-pragmatic group”: *M* = 8.2, *SD* = 2.9).

## Discussion

### Summary of Results

This study examined the narrative ability of cognitively-able children with and without ASD using the *Tuesday* picture book from the ADOS diagnostic battery. As hypothesized, the most pronounced difference between the ASD and TD groups was found in the semantic-pragmatic analysis. The ASD participants demonstrated reduced references to semantic-pragmatic elements, including basic story details (characters, settings, and actions) and complex concepts reflected in the story’s central ideas, as compared with their TD controls. This group difference was noted for ideas that related to ToM as well as to non-ToM concepts. The percentage of evaluative details and unrelated utterances did not differ between the groups. Analysis of additional narrative variables revealed that the TD group was more talkative as they produced generally more utterances than the ASD group, but the percentage of complex sentences in the narratives was higher for the ASD group. The last finding was somewhat surprising, but it nevertheless confirmed that the research group had overall language skills which were age-appropriate, and consequently any group effects that were found in their narrative skills could not be attributed merely to their syntactic performance. Morphological and syntactic errors were negligible in both groups, although the ASD group had more errors of this type. Taken together, the findings of the current study show that cognitively able children with ASD acquire the syntactic structures that are needed for the construction of a narrative. However, results of the detailed semantic-pragmatic analysis point to a semantic deficit in ASD. This difficulty in comprehending and relating to the variety of concepts that comprise the content of the story limits their ability to use their structural strengths and reduces the resulting narrative performance. The idea that semantics-pragmatics make up a core impairment in the narratives of children with ASD was supported by the significant negative correlation found between the number of central ideas expressed in the narratives of children with ASD and the ADOS-CSS-SA severity level. Thus, the more severe the social affect domain symptoms, the fewer references to central ideas in the *Tuesday* narratives. It should be noted, that although group differences were found in relation to semantic-pragmatic skills, an individual analysis revealed the heterogeneity of the ASD group. There is a sub-group of children with ASD whose performance was not different from the TD group.

The semantic-pragmatic analysis used in the current study was developed in order to target the central ideas that comprise the *Tuesday* story. Looking into the significance of each single central idea revealed that only two ideas differentiated the groups in favor of the TD group: “Frogs are flying over lily pads” (no. 1) and “The changing role of the dog” (no. 7). Interestingly, central idea no. 7 was also the only one that was included in over 75% of the narratives of the TD participants. However, because of the great variability among individual children within both groups, defining the number of expressed ideas in ranges appears to be a more practical measure (Table 5). Generally, 75% of the TD participants included more than three central ideas in their narratives, as compared with only 37.5% of the ASD participants.

### Theoretical Accounts

The semantic-pragmatic impairment expressed in the narrative performance of children with ASD can be linked to the neurocognitive differences suggested by the WCC account of ASD. The WCC account can explain the difficulty of the ASD participants to relate to the story’s details and central ideas. Regarding the story’s details, the ASD group included fewer characters, settings, and actions, but an equivalent number of objects, as compared with the TD group. This pattern is consistent with a WCC account because the objects in the *Tuesday* story are concrete and easy to interpret from the pictures (e.g., sandwich, window, cars). All other types of the story’s details are more abstract, and some of them require the integration of details in order to formulate accurate concepts [e.g., “police officer” (character), “living room of the old lady” (setting), “frogs chasing dog” (action)]. The central ideas require a global perspective for the creation of unified, higher-level concepts. Children need to correctly interpret a variety of details depicted in the pictures in order to infer the overall meaning of the scene.

Picture number 13, which depicts the police investigation, may illustrate the operation of this cognitive process in the *Tuesday* story. For this picture, the participant must integrate details regarding the man’s clothing, position, and facial expression, in order to infer that he is some kind of a detective (character) who is puzzled by the presence of the lily pads on the road (ToM central idea no. 10); the view of the road, cars, and pads in order to infer that this is a street in the city visited earlier in the story by the frogs (setting); the clothing and body language of the man in the rear of the picture, in order to infer that he is the man who was eating supper earlier in the story (character) and is now telling the investigators what he witnessed as they view the scene (action); and all the aforementioned specifics in order to formulate the central idea that the picture is describing a scene in which the police officers are trying to figure out what happened in their city last night (central idea *no. 9*). It is important to note, however, that the equal number of objects found in the narratives of the two groups does not support the reported superiority shown by individuals with ASD on tasks that require focusing on features, as postulated by the WCC account (Jolliffe and Baron-Cohen, 1997; Happé, 1999).

A second cognitive theory of ASD is the ToM deficit account. We analyzed separately the ToM- and non-ToM-related central ideas in order to look more closely at the ToM skills of the participants. However, the performance on both types of central ideas was lower in ASD as compared with TD. Therefore, a conclusion regarding a specific ToM impairment in addition to the generalized difficulty with abstract concepts cannot be supported by the findings of the current study. Finally, the third theoretical account of ASD, the EF impairment account, could not be supported by the findings of the current study either. The measure of unrelated text was taken to reflect one of the main EFs – the ability of the narrator to inhibit responses which are not directly related to the task at hand. No significant group difference was found in relation to this measure. Perhaps focusing on other EFs, such as working memory or planning, would result in significant group differences (Joseph and Tager-Flusberg, 2004).

### Previous Studies

Our major finding, the reduced ability of children with ASD to relate to the story’s main details and central ideas, agrees with findings of some of the previous studies, in which the *Tuesday* picture book from the ADOS was used for narrative elicitation (Rumpf et al., 2012; Banney et al., 2014; Suh et al., 2014; Kauschke et al., 2016; Kuijper et al., 2016). However, Rumpf et al. (2012) and Kauschke et al. (2016) did not find group differences in the overall references to *characters*, *time*, and *space*. The older and wider age range of the participants in these studies (8;0–19;0) (Rumpf et al., 2012) as compared with the participants in the current study (4;10–7;0) may explain this difference in results. At an older age, children with ASD may have acquired the ability to relate to these basic story elements. Nevertheless, previous studies looked mostly at the explicit semantics by analyzing characters and events in the story. The only study that listed a variety of story’s events (Suh et al., 2014) focused mostly on the description of the picture in each page of the book. The current study added global, more abstract semantics to these measures. The elaborated coding system for the story’s semantic-pragmatics included both concrete and abstract concepts, and differentiated ToM from non-ToM related ideas. It included 6 different settings, 7 characters, 8 objects, 11 actions, and 12 abstract central ideas. This unique, detailed analysis enabled a comprehensive look into the children’s semantic-pragmatics and enabled the identification of more subtle differences between children with and without ASD. Thus, the findings of the current study point to a semantic-pragmatic deficit in expressing story ideas among children with ASD in the age range of 4;10–7;0 (the youngest age range for using ADOS Module 3) who otherwise show age-appropriate cognitive and linguistic functions.

Verbal productivity, measured in length of the produced narratives, yielded group differences in the current study and in Rumpf et al. (2012) and Kuijper et al. (2016). Banney et al. (2014), Suh et al. (2014), and Kauschke et al. (2016) found equivalent story lengths for their groups. Once again, the different age ranges of participants in these studies may explain this inconsistency. It is possible that younger children with ASD produce shorter narratives than children with TD, but with time, the ability to narrate and describe visual stimuli matures, and helps them produce narratives of equivalent length.

The current study found that the ASD group made a greater number of errors, but produced a higher level of syntactic complexity than the TD group in their narratives. The greater number of errors made by the ASD group should be viewed within the context of a low percentage of errors overall. Rumpf et al. (2012) did not find group differences in any syntactic complexity measures. Banney et al. (2014), on the other hand, found that the narratives of children with ASD were syntactically less complex but included an equivalent number of grammatical errors. The ASD group in Kuijper et al. (2016) produced more errors and used less complex syntactic structures as compared with the TD group. The higher level of syntactic complexity found in the current study may be explained by the strict cognitive criterion that was used in the ASD group. All children with ASD had to achieve a score of over 85 on both the verbal and the non-verbal sections of the WPPSI-III in order to be included in the study. This criterion may have screened out children with lower syntactic functioning.

The average number of *evaluative comments* was not found to differentiate the groups in the current study, which is consistent with the findings of Rumpf et al. (2012); Banney et al. (2014), and Kauschke et al. (2016). Likewise, the current study did not find a group difference in relation to utterances that were not directly related to the story. Our measure included associative comments and descriptions that pointed to an inaccurate interpretation of the story’s pictures. In Suh et al. (2014), a measure of “idiosyncratic language and unusual references” was defined as language that is used in an unconventional manner, such as overly formal speech, scripted language, or made-up words. The researchers found that more participants with ASD used idiosyncratic language than their TD peers. An interesting finding in the current study is related to the relatively high percentage of unrelated utterances that were produced by the TD participants. This finding suggests that imaginative stories such as *Tuesday* may be difficult for many children aged 5;0–7;0 to interpret, and this may reduce their ability to fully express their narrative competence.

### The Normative Performance on the *Tuesday* Story

In addition to the identified deficits in the narrative competence of children with ASD, the findings of the current study shed an important light on the normative performance of young TD children. To date, data regarding the characteristics of narratives produced by TD children of the *Tuesday* story have not been available to researchers and clinicians. The developmental literature points to significant gains in the narrative capacity of children 5;0–7;0 years old (e.g., Berman and Neeman, 1994). The *Tuesday* storybook is part of the ADOS diagnostic battery, and it is important to know what can be expected of TD children in the specified age range. Looking at the numbers of items in each category expressed by the TD participants, it can be seen that children between 5;0–7;0 years of age are capable of including in their *Tuesday* narratives about 80% of the characters, 60% of the actions, 40% of the objects, 40% of the settings, and 30% of the story’s central ideas, as detailed in this study’s analysis scheme.

### Implications of the Current Study

The current study has important clinical implications. When using Module 3 of the ADOS in the ASD diagnosis procedure with young children, in addition to the formal scoring of the ADOS, clinicians might benefit from focusing on those measures that clearly distinguish between the performance of cognitively able children with ASD and children with TD. The distinctive measures in the age range of 4;10–7;0 years appear to relate to the story’s semantic-pragmatics, both at the concrete and the abstract levels. According to the current study, distinctive measures do not appear to include syntactic complexity or evaluative comments. In addition, the association found between the severity of social affect deficit and the number of central ideas and *characters* points to the possible use of these measures in evaluating the severity of the ASD symptoms. Since most central ideas of the *Tuesday* story appeared to be difficult for children 4;10–7;0 years old (both with ASD and with TD) to express (a finding which needs additional support from research), using the simpler story Good Night, Gorilla (Rathmann, 1996) from the ADOS Module 2 with young, linguistically competent children should be considered. The finding regarding a semantic-pragmatic deficit in the current study should encourage clinicians to focus on these aspects when other stories are used, both in diagnosis procedures and during intervention. Perhaps a list of each story’s central ideas can be compiled in advance, and narratives can be analyzed accordingly.

The individual differences within the ASD group regarding narrative semantic-pragmatic skills reflect the known heterogeneity of this clinical population. Children with ASD are heterogeneous in their cognitive and language skills, and also in their narrative semantic-pragmatics. Some children perform below the mean of their age-matched controls, while others perform just as well as their TD peers. The clinical implication of this heterogeneity is that children with ASD should be assessed individually on this narrative aspect, and treatment should be provided to those children who have a difficulty in this area.

The current study adds novel information regarding the narrative performance of children with ASD in several ways. The age range of participants was narrowed to 4;10–7;0 years, which is the youngest age range for which the ADOS Module 3 can be administered. The analysis was comprehensive and included measures of central ideas that reflect global meanings that might require more than one cognitive operation. In the current study, the basis for analysis was the unique ideas present in each page of the *Tuesday* story. Finally, this is the first study to demonstrate the association between narrative semantics and autism severity as measured with standardized tests.

### Strengths and Limitations

The current study has several strengths. The participants with ASD underwent a comprehensive diagnosis based on the widely used diagnostic tools (ADOS and ADI-R). The inclusion criteria were strict regarding cognition, both verbal and non-verbal. The research group was relatively large, as compared with previous studies, and the age range of participants was relatively narrow, which strengthened the homogeneity of the group.

The current study has a few limitations that should be considered. Only two cognitive subtests were used with the TD participants. In addition, the participants in both groups did not undergo a comprehensive language evaluation, which could have provided additional information about their lexical and syntactic capacities.

### Future Research

Future research could use the elaborated coding system developed for the current study in older children, among whom an overall higher level of narrative performance is expected. It would also be interesting to investigate whether presenting participants with the pictures prior to telling the story (a different procedure than the one used in the current ADOS protocol) improves their performance and better demonstrates their competence, by providing essential clues to the story’s main ideas. In order to more specifically measure participants’ comprehension of the story’s central ideas, future research should consider the assembly of a set of specific questions to present to the participants either during or after their narrative production. The current study may provide a basis for future studies to address narrative development in children with ASD, taking into consideration the cognitive accounts of ASD (ToM deficit, EF deficit, WCC), and language skills of children with ASD. Specific measures related to the three theoretical accounts discussed should be used with participants and analyzed within correlational tests, in order to find direct relationships between the relevant cognitive functions and narrative measures. The notion of high- and low- narrative semantic-pragmatic-subgroups in ASD might follow in the tradition of profiling children with ASD based on their verbal or cognitive skills. Although the current study could not fully support this notion because of the relatively small ASD populations that would have resulted from the division of the ASD group into these two subgroups, it may be the first step for future studies to examine this hypothesis. Future studies should attempt to look more closely into this cognitive-linguistic aspect and its associations with other cognitive and linguistic skills.

Finally, the elaborated central ideas coding system should be adjusted and tested with additional stories, other than *Tuesday*.

## Data Availability Statement

The datasets generated for this study are available on request to the corresponding author.

## Ethics Statement

The studies involving human participants were reviewed and approved by the Institutional Review Board (IRB) of Ariel University. Written informed consent to participate in this study was provided by the participants’ legal guardian/next of kin.

## Author Contributions

All authors listed have made a substantial, direct and intellectual contribution to the work, and approved it for publication. NK, DZ, and EB-I worked on design, data collection, data analysis, and writing. LW contributed in inferring the study’s conclusions and in the writing.

## Conflict of Interest

The authors declare that the research was conducted in the absence of any commercial or financial relationships that could be construed as a potential conflict of interest.
