# Supplementary material for: Semantic-Pragmatic Impairment in the Narratives of Children With Autism Spectrum Disorders
Source: Front Psychol. 2019 Dec 12;10:2756. doi: 10.3389/fpsyg.2019.02756 (PMC6920157; doi:10.3389/fpsyg.2019.02756)
Supplement: Supplementary file 1 [file Data_Sheet_1.pdf]

Appendix A: Studies on narrative skills in ASD

Table A1: Selected studies

Study	Participants	Measures	Results
Diehl, Benetto & Young, 2006[18]	ASD (n=17) Mean age = 8;10	**Task: story Retelling**	
	TD (n=17) Mean age = 9;6	Length	ASD=TD
		Syntactic complexity	ASD=TD
		Story events	ASD=TD
		Coherence	ASD<TD
Norbury & Bishop, 2003[19]	ASD (n=12) Mean age = 8;10	**Task: picture book**	
	TD (n=18) Mean age = 8;7	Narrative structure	ASD=TD
		Length	ASD = TD
		Syntax	ASD<TD
		Semantic score	ASD=TD
		Cohesion	ASD<TD
		Evaluation	ASD=TD
Norbury & Bishop, 2002[20]	ASD (n=10) Mean age = 8;11	**Task: story recall**	
	TD (n=18) Mean age = 8;8	Recall score	ASD=TD
		Comprehension score	ASD<TD
		Error responses	ASD>TD
Novogrodsky, 2013[21]	ASD (n=24) Mean age = 10;0	**Tasks: storytelling and story**	
	TD (n=17) Mean age = 9;10	**retelling**	ASD = TD
		Length	ASD = TD
		Complex sentences	ASD = TD
		3rd persons pronouns	ASD>TD on story
		Ambiguous pronouns	telling

Losh & Capps, 2003[22]	ASD (n=28) Mean age = 11;4<br>TD (n=22) Mean age = 10;7	**Tasks: storytelling and personal narratives**<br>Grammatical complexity<br>Evaluation<br>Syntactic diversity<br>Causal explanations<br>Structure<br>Prompts<br>Irrelevant remarks	 <br>ASD=TD / ASD<TD<br>ASD=TD / ASD<TD<br>ASD=TD / ASD<TD<br>ASD<TD / ASD<TD<br>ASD<TD<br>ASD>TD<br>ASD>TD
Makinen, Loukusa, Leinonen, Moilanen, Ebeling & Kunnari, 2014[23]	ASD (n=16) Mean age = 7;7<br>TD (n=16) Mean age = 7;5	**Task: picture book**<br>Length (C units & NDW)<br>Syntactic Complexity<br>Referential Accuracy<br>Story details (29 units)<br>Additional information<br>Irrelevant information<br>Mental state expressions<br>Comprehension	 ASD<TD<br>ASD=TD<br>ASD=TD<br>ASD<TD<br>ASD<TD<br>ASD>TD<br>ASD=TD<br>ASD<TD
Sah & Torng, 2015[24]	ASD (n=18) Mean age = 8;2<br>TD (n=18) Mean age = 7;0	**Task: picture book**<br>Length<br>Causal connectives<br>Causal networks	 ASD=TD<br>ASD=TD<br>ASD<TD

ASD = Autism Spectrum Disorders, TD = Typical Development

Table A2: Previous ADOS Tuesday studies

Study	Participants	Measures	Results
Banney, Harper-Hill, & Arnott, 2014[28]	ASD (n=11)<br>TD (n=17)<br>Age range: 9;0-15;2	Length<br>Fluency<br>Errors<br>Lexical Diversity<br>Syntactic complexity<br>Syntactic diversity<br>Ambiguous pronouns<br>Episodic structure<br>Two core events<br>Evaluation<br>Emotional & cognitive terms	ASD = TD<br>ASD = TD<br>ASD = TD<br>ASD = TD<br>ASD<TD<br>ASD = TD<br>ASD>TD<br>ASD<TD<br>ASD<TD<br>ASD = TD<br>ASD = TD
Kuijper, Hartman, Bogaerds-Hazenberg, & Hendriks, 2016[29]	ASD (n=36)<br>TD (n=36)<br>Age range: 6;1-12;10	Verbal productivity (MLU)<br>Fluency (Repetitions)<br>Syntactic complexity (Clauses)<br>Syntactic complexity (Errors)<br>Lexical diversity<br>Emotional & cognitive terms<br>Referencing<br>Causal conjunctions	ASD<TD<br>ASD>TD<br>ASD<TD<br>ASD>TD<br>ASD = TD<br>ASD = TD<br>ASD = TD<br>ASD<TD
Rumpf, Kamp-Becker, Becker, & Kauschke, 2012[30]	ASD (n=11)<br>TD (n=11)<br>Age range: 8;0-12;11	Length<br>Gramm. complexity<br>Temporal/Causal conjunctions<br>Coherence – referencing<br><br>2 main events<br>Speaker's perspective	ASD<TD<br>ASD = TD<br>ASD = TD<br>More explicit less<br>implicit<br>ASD<TD<br>ASD = TD

		Narrative style<br>Internal state language	ASD = TD<br>ASD<TD
Suh, Eigsti, Naigles, Barton, Kelley, & Fein, 2014[31]	ASD (n=15)<br>TD (n=15)<br>Age range: 9;9-15;7	Length<br>Lexical diversity<br>Story elements (events)<br>Pronoun use<br>Fluency (Repetitions)<br>Mental state expressions<br>Causal conjunctions<br>Idiosyncratic language	ASD = TD<br>ASD = TD<br>ASD<TD<br>ASD<TD<br>ASD>TD<br>ASD = TD<br>ASD = TD<br>ASD>TD
Kauschke, van der Beek, & Kamp-Becker, 2016[32]	ASD (n=22, 11 boys & 11 girls)<br>TD (n=11)<br>Age range: 8;0-19;0	Length<br>Coherence<br>Cohesion (including references to characters, time, space, and 2 core events)<br>Evaluative Devices<br><br>ISL (emotion, cognition, physiology, evaluation, modality, causality)	**Girls: ASD vs. TD**<br>ASD = TD<br>ASD = TD<br>ASD = TD<br><br>ASD = TD<br><br>ASD<TD on emotion words

ASD = Autism Spectrum Disorders, TD = Typical Development

Appendix B: Transcripts

Child with ASD

Picture	Examiner	Child
1	Once upon a time at 8:00 p.m., the turtle stood on a log in the swamp and looked up into the sky, when suddenly….. Now you look and continue the story…	
2		One evening, very late at night, a frog came out and went and went for a walk in the city on a nice rounded leaf. Now it's your turn!
3	You keep going!	
4		
5	Keep going a little more.	And suddenly one man was eating supper. And he saw that the frogs… Now you!
6		And one night the wind came and blew off their blankets. They got in the blankets and they flew away. They tied small kerchiefs to themselves.
7		One day they got into old grandma's home.
8		
9		And suddenly one frog was chasing them.
10		Now the frogs too were chasing him.
11		**One day the frogs flew away**.
12	And suddenly the morning came and the frogs flew back to their swamp. Now you!	
13	It is the police!	And then all the people walked on the path and went to their work, and he was going to work, this is a military car.<br><br>**The police are looking**, he is a policeman also. He always drives this car. A policeman's car. They went up; this man went on a truck. Now you!
14		One day the evening had come, and the rhinoceros went to sleep.
15		Except for the bat that stayed awake.

Child with TD

Picture	Examiner	Child
1	Once upon a time at 8:00 p.m., the turtle stood on a log in the swamp and looked up in the sky, when suddenly….. Now you look and continue the story…	
2	Nice!<br>That's right! You tell me what you see in the picture.<br><br>Yes, he was startled.	And then a frog jumped on a leaf. And two more frogs.<br>That's in the picture.<br><br>**And the turtle was startled**!
3	Yes, it looks like they are having fun.	And the **frogs flew on a leaf**, and they had fun.<br>And here they didn't have fun, and later it started being fun for them.
4		The frogs flew over the houses, and they sat down and looked at the houses.
5	He was a little shocked. That's right.	And one man was eating bread, and he looked at the frogs, and **he was a little shocked**.
6	And what?<br><br>That's right.	And the frogs, suddenly, the fabric got into the eye of one of them…<br><br>There was wind and the blanket flew over the frog's face.<br>And it was not fun!
7	Very nice. Keep going. Good for you!	Later they got into a house, and others got in through the window.
8		Later they flew again and there were frogs in the house, many frogs, and **the woman did not notice**, and the frogs were watching television.
9		Suddenly they flew, and suddenly the wolf came to eat them. He was chasing her.
10		All the frogs **later on chased him**. He ran away, ran away.
11		Suddenly they flew in the wind. One of them did not have fun. The other one did have fun.
12		At the end they fell into the pond.<br>It's not the whole story.<br>It's a long story.<br>They jumped into the pond, and then they jumped on their leaves.

13	Who is this man?	The policemen saw the leaves and one man said that there were flying frogs.<br><br>The one talking on the microphone here.
14	Wait a second, I forgot to tell you that here it is a week later, a week later – what happened?	And there was… it's in a little cabin, the wolf.<br><br>A week later there was a wolf in a little cabin.
15	You told the story nicely!	And later chickens and pigs jumped out of the cabin.<br>And the story ends. Really.

Note: Expressions of central ideas are in bold.
